# Does drug treatment improve patient quality of life? A pilot study of the outcomes of the quality of life assessment in New York City outpatient and opioid treatment programs

**DOI:** 10.1186/1940-0640-10-S1-A43

**Published:** 2015-02-20

**Authors:** Mindy D Nass, Anne E Siegler, Lara Maldjian, Luke Bergmann, Hillary V Kunins

**Affiliations:** 1 New York City Department of Health and Mental Hygiene, Queens, New York, 11101, USA

## Background

The Affordable Care Act and the Medicaid redesign in New York City offer opportunities to explore alternative methods for measuring the effectiveness of behavioral health interventions. Quality of life (QOL) measures have been underutilized in substance use disorders treatment (SUDT). The objective of this study was to determine how a validated QOL instrument could be used in SUDT as a measure of health-related patient outcomes.

## Methods

NYC outpatient drug treatment (DT) and opioid treatment programs (OTP) were invited to participate in a pilot evaluation. Newly admitted patients completed counselor-administered surveys at admission, and 90 and 180 days. Surveys included demographic (gender, age, race, language) and clinical items (homelessness, criminal justice involvement, mandated treatment, major health conditions, substance of choice, frequency of use) in addition to the World Health Organization QOL instrument, the WHOQOL-BREF [1]. The WHOQOL-BREF is a 26-item, validated questionnaire that measures QOL in four domains: physical, psychological, social, and environment. Domain-specific QOL scores were calculated, transformed, and compared with healthy and chronically ill populations from the literature [2]; scoring was on a scale of 0 to 100 for each domain. We compared mean domain scores between baseline and follow-up intervals for available participants, and by demographic and clinical characteristics using ANOVA and *t*-tests. We examined change in QOL scores among OTP participants stratified by major health conditions.

## Results

**Figure 1:**
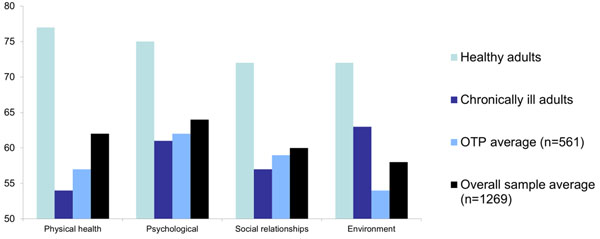
Comparison of baseline QOL domain scores in SUD population to healthy and chronically ill U.S. adult populations in the literature [2]

**Figure 2:**
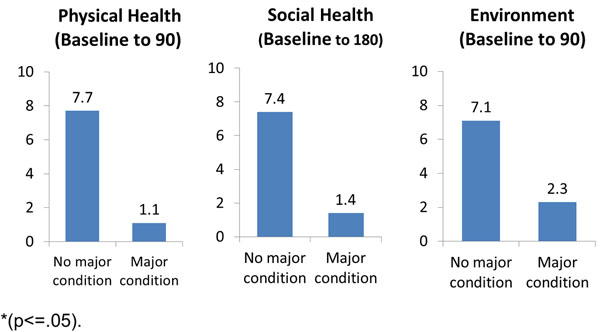
Change in 3 QOL domains over time among OTP participants by major health conditions*

Between July and September 2013, 1269 newly admitted patients were surveyed. Follow-up surveys were completed for 616 patients at 90 days (49%) and 336 at 180 days (26%). See Table 1 for demographic characteristics. NYC SUDT participants had lower mean QOL scores in the physical and psychological domains than healthy U.S. adults, and higher than chronically ill U.S. adults [2] (Figure 1). Mean QOL scores increased over time for all domains (Table 2). OTP participants with major health conditions had smaller increases in QOL compared to OTP participants without major health conditions (Figure 2).

**Table 1:** Participant Demographic Characteristics (N = 1269)

Demographic characteristic	n	%
Female	354	28
Race		
White	317	25
Black	470	37
Hispanic	431	34
English language	1103	84
Under age 45	596	47
Homeless	292	23
Any criminal justice involvement	302	24
Major physical or mental health condition	736	58

**Table 2:** Domain-specific mean QOL scores at baseline, 90, and 180 days*

**Domain**	Baseline (n = 1262)	90 Day (n = 616)	180 Day (n = 336)
Physical	62	64	66
Psychological	64	65	69
Social	60	61	64
Environmental	58	60	63

* *T*-tests were used to examine differences in mean domain scores between baseline and 90 days, and between baseline and 180 days. Changes in mean QOL scores were significant from baseline to 90 days (p < .05) and from baseline to 180 days (p < .05) in all domains.

## Discussion

Preliminary findings indicate that individuals in SUDT have lower QOL scores than healthy populations and experience improvements in QOL during treatment. Small positive changes among individuals with health conditions suggest the importance of integrating physical health care with SUDT. High dropout rates, multiple survey administrators, and an English-only survey instrument may limit our conclusions. Future investigations need to examine the feasibility of incorporating QOL measures into SUDT more broadly, including its impact on clinical interventions and longer-term patient outcomes, including maintenance of QOL gains achieved during SUDT.
